# Neotropical and North American Vaccinioideae (Ericaceae) share their mycorrhizal Sebacinales - an indication for concerted migration?

**DOI:** 10.1371/currents.RRN1227

**Published:** 2011-03-03

**Authors:** Sabrina D. Setaro, Kathleen A. Kron

**Affiliations:** Department of Biology, Wake Forest University, 1834 Wake Forest Road, Winston-Salem, NC 27106, USA

## Abstract

Neotropical Vaccinioideae (Ericaceae) are evolutionarily rather young and presumably of Northern Hemisphere origin. Vaccinioideae are highly dependent on their mycorrhizal symbionts and Sebacinales (basidiomycetes) were previously found to be the dominant mycobionts of Andean Clade Vaccinioideae (Neotropical Vaccinieae). We were interested to see whether the North American Vaccinioideae reached the Neotropics with their mycobionts or whether they acquired new, local Sebacinales.

We investigated Sebacinales of 58 individuals of Vaccinioideae from Ecuador, Panama and North America to examine whether mycobionts of each region are distantly or closely related.

We isolated the ITS of the ribosomal nuclear DNA in order to infer a molecular phylogeny of Sebacinales and to determine Molecular Operational Taxonomic Units (MOTUs). MOTU delimitation was based on a 3% threshold of ITS variability and conducted with complete linkage clustering. The analyses revealed that most Sebacinales from Ecuador, Panama and North America are closely related and that two MOTUs out of 33 have a distribution ranging from the Neotropics to the Pacific Northwest of North America. The data suggest that Neotropical and temperate Vaccinioideae of North America share their Sebacinales communities and that plants and fungi migrated together.

## Introduction

Neotropical Vaccinioideae are a highly diverse group of Ericaceae [1]. Most of the species diversity of Vaccinioideae is found in the tribe Vaccinieae, especially in the Andean Clade. However, there are also many Neotropical species of the widespread tribe Gaultherieae [1]. The current hypothesis is that the ancestors of Neotropical Vaccinioideae are of North American origin and that there were at least two dispersals into South America leading to diversification of Vaccinieae and portions of Gaultherieae [2]. During the uplift of the Andes, the Vaccinieae are believed to have undergone rapid radiation which led to its high species diversity [2]. Some Andean Clade species presumably colonized Mesoamerica and other regions in South America forming the distribution range we see today [2]. Currently, many Neotropical Vaccinioideae are endemic in the Northern Andes [1].

All Ericaceae are mycorrhizal and highly dependent on their mycobionts [3] [4]. The mycobionts mostly belong to the *Rhizoscyphus ericae* aggregate (ascomycetes), are species of *Oidiodendron* (ascomycetes) or basidiomycetes of the order Sebacinales [4] [5] [6] [7]. Most research on mycorrhizae in Ericaceae was conducted on ericoid mycorrhizal Ericaceae [8] [9] [10] [11] [12], a mycorrhizal type formed by all ericaceous subfamilies except Enkianthoideae, Arbutoideae and Monotropoideae [4]. It has been shown that due to their mycobionts, Ericaceae are able to dominate harsh environments with soils of low nutrients and high acidity [3] [4] [13]. Mycorrhizal studies during the last decade revealed that Sebacinales are common mycorrhizal fungi of Ericaceae [6] [7] and that they are the dominant mycobionts of Andean Clade Vaccinieae [14] [15].  With this study we propose to shed light on the evolutionary history of Neotropical Vaccinieae and Gaultherieae and their mycorrhizal Sebacinales. We test two hypotheses based on the fact that Ericaceae are dependent on their mycorrhizal fungi [3] [4] and the assumption that Neotropical Vaccinioideae are of North American origin [2].

The first hypothesis assumes that migration of ancestral Vaccinieae and Gaultherieae was coupled to the presence of their established mycobionts. Mycorrhizal fungi are transmitted horizontally by colonizing the host plant from the surrounding soil and are not vertically transferred from one generation to the next [16]. Therefore, we suggest that such a migration would be rather slow since it would depend on the dispersal capacities of both plants and fungi. Long distance dispersal of propagules is highly unlikely, since it would require a co-transport of seeds and spores. If the hypothesis of a coupled migration is true, we expect to find Sebacinales from North America, Mesoamerica and South America to be closely related and Sebacinales of Neotropical Vaccinioideae to be placed in more derived clades.

Alternatively, if ancestral Vaccinieae and Gaultherieae migrated independently of their established mycorrhizal community and the plants associated with local Sebacinales, we expect to find different clades of Sebacinales in each region, i.e., North America, Mesoamerica and South America. Such an independent migration may have occurred by long distance dispersal of the plants in a relatively short time span.

Our knowledge of Sebacinales associated with Neotropical Vaccinioideae is based on samples from a relative small area in Southern Ecuador [7] [14] [17] [18]. We know that Sebacinales of Neotropical Vaccinioideae belong to several clades that in most cases, to date, do not occur anywhere else. However, to estimate the species distribution range of these Sebacinales, we need more samples from other Neotropical areas. Furthermore, phylogenetic inferences of Sebacinales were predominantly based on LSU (ribosomal large subunit) sequences of the D1/D2 region. The LSU is rather conservative [19] and may therefore not be appropriate for the resolution of Sebacinales at species level and hence the estimation of distribution ranges. The ITS (ribosomal internal transcribed spacer), however, is more variable and is considered a good target for barcoding of basidiomycetes [20] [21] [19].

In fungi and other cryptic organisms, delimitation of molecular operational taxonomic units (MOTUs) allows the estimation of diversity and distribution as well as the comparison of different hosts and vegetations [19] [22] [23] [24] [25]. A threshold of 3% ITS variability is commonly applied in fungi, because it seems to be an appropriate estimate for species delimitation [19]. At present this is the only option for defining Sebacinales, because many species do not form fruiting bodies [26] [27], their cultivation on agar plates is challenging [6] [28] and they lack distinguishing morphological characters on sterile hyphae [26] [27] [29].

To test our hypotheses we expanded the existing ITS Sebacinales data set by the inclusion of 86 sequences isolated from several Neotropical Vaccinieae and Gaultherieae from Ecuador, Panama and North America. We performed phylogenetic analysis and complete linkage clustering on the basis of 3% ITS variability to delimit MOTUs of Sebacinales associated with Ericaceae. 

## Materials and Methods

### Study sites

Mycorrhizae of Vaccinioideae were collected in the following locations:

Southern Ecuador - Reserva Biológica San Francisco (for detailed habitat description see [15] [30], mycorrhizae from 31 plants. Southwest Panama - Alto Chiquero, Bajo Mono, La Fortuna, Parque Internacional de La Amistand, Volcano Barú, 2008, mycorrhizae from 20 plants. Pacific Northwest of North America - Mount Baker, Whidbey Island, 2008, mycorrhizae from 6 plants. Southeast North America - Linville Gorge, 2007, mycorrhizae from 1 plant. In total we collected mycorrhizae from 58 plant individuals (tab. 1a,b).

Google Maps

### Molecular Approach

Genomic DNA was isolated from dried mycorrhiza samples (~5mm root length per host individual) using the DNeasy Plant Mini Kit (Qiagen), according to the manufacturer’s instructions but without the use of RNase A. To amplify the ITS of the nuclear ribosomal DNA, we used the universal forward primers NS23 [27] and ITS1F [28] as well as the Sebacinales-specific reverse primer NLSeb2R [31] in polymerase chain reactions (PCRs). The genomic DNA extract with a final concentration of 0.1 was the template for the PCR reaction. In some cases, a nested PCR was required to obtain products suitable for cloning. The first amplification was conducted with the primers NS23/ITS1F and NLSeb2R. This product was used as template with final concentrations of 0.02, 0.002 or 0.0002 for a second PCR with the primers NS23/ITS1F and the Sebacinales-specific primer NLSeb1R [31]. We used the Phusion Master Mix with HF buffer (Finnzymes) as PCR reagents with a final volume of 20 µl. The PCR protocol was followed according to the manufacturer’s instructions, but with 30 cycles and an annealing temperature of either 60° C, 63° C or 66.5° C.

All positive PCR products were cloned with the Zero Blunt Topo PCR Cloning Kit (Invitrogen, Life Technologies) using a PCR product volume of 0.5 µl for the cloning reaction. Clones were checked for positive inserts by picking one to ten bacterial clones, diluting them in ddH_2_O (ten or twenty fold, depending on the size of the colony) and placing them in a PCR reaction mix. The PCR was conducted with Taq polymerase (either Invitrogen, Life Technologies or GoTaq Colorless Master Mix, Promega), M13F and M13R as primers and final clone concentration of 0.005 or 0.01. One to ten positive clones were either amplified by rolling circle amplification with the TempliPhi Amplification Kit (GE Healthcare) and sequenced without prior purification on an ABI 3730 sequencing machine (GATC Biotech, Germany) or the positive inserts were purified with ExoSAP-IT (0.05% - USB Corporation, USA) and sequenced on an ABI 3130 sequencing machine (Medical Center WFU, USA). In addition to M13F and M13R, we used several primers (NS23; ITS3Seb: 5’-TGAGTGTCATTGTAATCTCAC-3' kindly provided by M. Berbee; LR3: [29]; LR0R: [29]; SSS1: 5'-GTGAACCTGCGGAAGGATCATTA-3'; MWS1; ITS1F; NL4 reverse complement: [30]; SSS2: 5'-TAGATG TTCTGGGCCGCACGC-3'; SSS3: 5'-GGAATAGGGAGAATCTGC-3') to obtain a full sequence length of good quality.

All sequences were edited and congruent strands were combined using Sequencher 4.6 (Gene Codes, Ann Arbor, MI, USA) or Geneious [32]. BLAST [31] against the NCBI nucleotide database http://www.ncbi.nlm.nih.gov/ was used to check the sequence identities. Sequences that matched Sebacinales were compiled in a data set and checked for potential chimeric parts. To this end, sequences were aligned with POA [32] and analyzed with Bellerophon [33] using a window width of 200 base pairs. In addition, the alignment was partitioned with a window width of 400 base pairs and each partition was blasted separately. All sequences have been submitted to Genbank (tab. 1a,b).

## Phylogenetic Analyses

We compiled a data set including our own Sebacinales sequences and ITS sequences of Sebacinales from ericaceous hosts available on Genbank. This data set was automatically aligned with POA [32], an alignment program especially suited for data sets with large indels [32]. Such indels are frequently found at the beginning of the ITS in Sebacinales (personal observation). For the sake of maximum reproducibility, no further manual corrections were made on the alignment. To obtain a consistent data set of only ITS and 5.8S data, we deleted all nucleotides belonging to the 18S and 28S ribosomal nucDNA. Complete linkage clustering with a threshold of 1% sequence dissimilarity on the ITS1- 5.8S-ITS2 was performed with OPTSIL [37] to prune redundant sequences from the data set. All sequences from the same cluster and isolated from the same host plant and locality were excluded with the exception of one representative. After pruning, the data set was realigned with POA and a Maximum Likelihood analysis was performed with 1000 rapid bootstrap replicates [35] in RAxML 7.0.4 sequential version using the GTRmix model [36]. Once the ML analysis was finished, the tree was midpoint rooted with FigTree [38]. The ITS tree and the respective alignment were submitted to TreeBASE http://purl.org/phylo/treebase/phylows/study/TB2:S11205.

## Delimitation of Molecular Operational Taxonomic Units (MOTUs) and analysis of richness

In order to delimit Sebacinales sequences to MOTUs, we used a threshold of 3% ITS1-ITS2 variability in combination with complete linkage clustering. A p-distance table including only ITS1-ITS2 data of all sequences was created with PAUP [39] and complete linkage clustering was performed with OPTSIL [37]. The complete linkage approach combines those sequences in a cluster that all have a p-distance below the threshold when compared to each other [37] [40]. This approach is likely to result in more clusters than e.g. single linkage where only one link is required to combine sequences in a cluster. We chose complete linkage over single linkage, because this approach is less likely to overestimate distribution range. In order to estimate richness of MOTUs in our sampling (data from North, South and Mesoamerica combined), we calculated a sample-based rarefaction accumulation curve with 95% confidence intervals using EstimateS v.8.2.0 [33] and the setting "randomization without replacement".

Table 1a: Accession number of all sequences obtained in this study with respective MOTU (Molecular Operational Taxonomic Unit) number (if applicable) and information about samples, hosts and localities. Accessions in bold indicate sequences used for phylogenetic analysis and delimitation of MOTUs after redundant sequences were excluded from the data set. Abbreviations: RBSF = Reserva Biológica San Francisco; PILA = Parque Internacional de La Amistad. 

**Figure:**
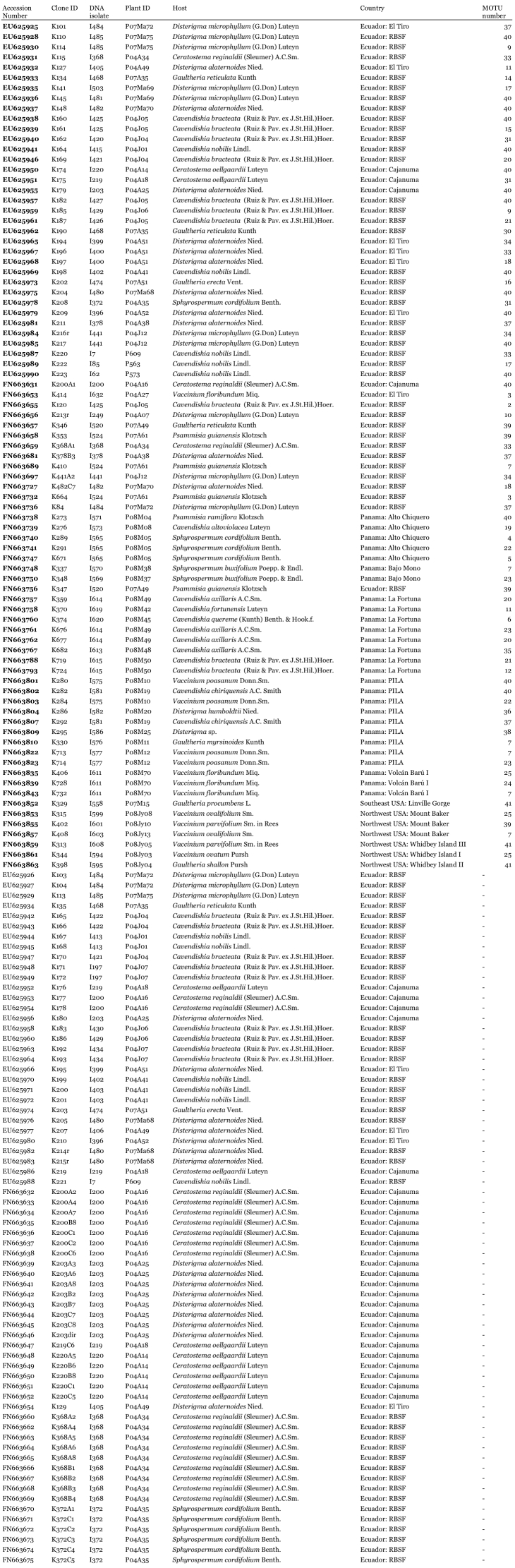

Table 1b: Continuation of table 1a.

**Figure:**
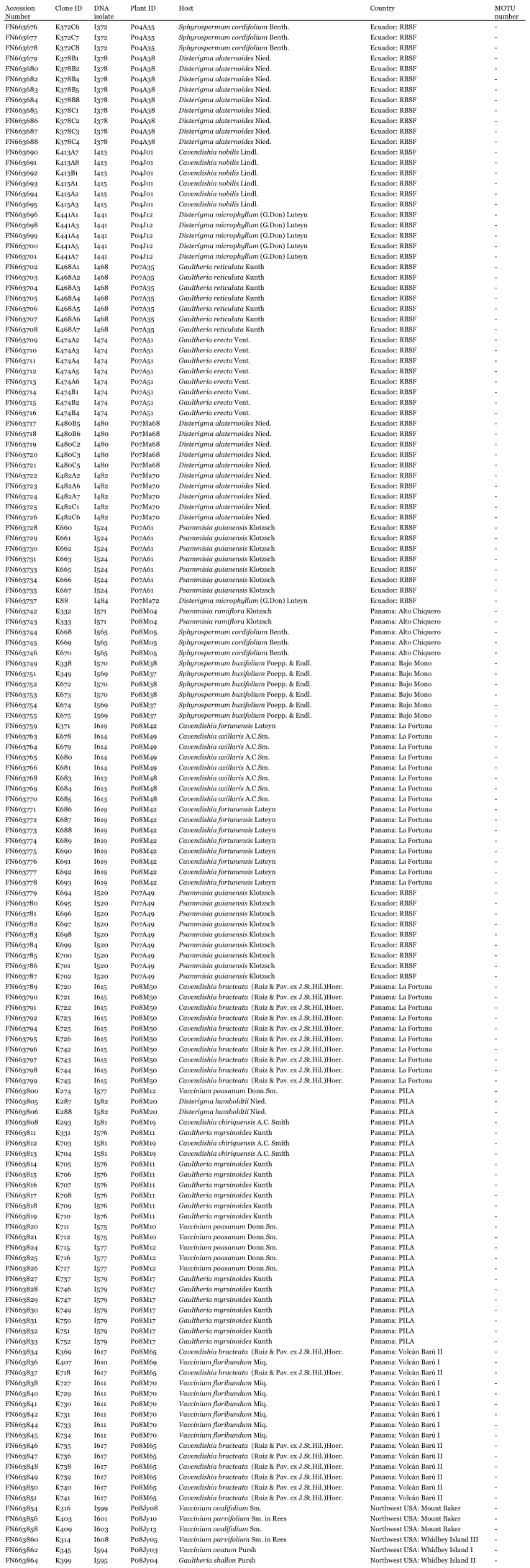

## Results

The Maximum Likelihood analysis based on ITS data revealed several supported clades showing the close relationship of Sebacinales associated with Ecuadorian, Panamanian and North American Vaccinieae and Gaultherieae (fig. 1). There are six major subclades containing Sebacinales from American Vaccinioideae with weak (50-69%), moderate (70-89%) or high (90-100%) bootstrap support (fig. 1). Subclade 1 is a well-supported group and consists almost entirely of Sebacinales associated with Neotropical Vaccinioideae with the exception of one accession that was associated with *Vaccinium parvifolium* from the Pacific Northwest of North America (fig. 1). Subclades 2, 3 and 6 contain Sebacinales only from Neotropical Vaccinioideae occurring in Ecuador and Panama. Subclade 4 consists of Sebacinales from Ecuador, Panama and North America (fig. 1). Subclade 5 shows the largest geographic range, since it contains Sebacinales associated with Vaccinioideae from Ecuador, North America and La Reunión (fig. 1). Sebacinales from Vaccinieae and Gaultherieae are closely related (clades I, III, IV, V, VI - fig. 1). However, none of the Sebacinales of American Vaccinioideae in this data set are closely related to Sebacinales associated with Ericaceae belonging to the subfamilies Arbutoideae (*Arbutus*) or Monotropoideae (*Pyrola, Orthilia*) (fig. 1).

The cluster analysis based on a threshold of 3% ITS1-ITS2 variability revealed 47 MOTUs in total, 27 of these are singletons and 35 occur in host plants collected for this study (fig. 1). The rarefaction accumulation curve, based on the samples from our data set, did not reach their asymptote and Jackknife 2 and Chao 2 indices estimate a richness of 50 to 60 MOTUs total. 

Most MOTUs correspond to clades, but four MOTUs (37, 38, 44, 45) occur in different clades (fig. 1). In the case of MOTU # 37 and 44, the splits are unsupported, but for MOTUs # 38 and 45 there are moderate bootstrap supports for each split (fig. 1). Twenty-eight MOTUs are endemic to either Ecuador or Panama (fig. 1). Six MOTUs (# 7, 20, 21, 37, 38, 40) occur in Ecuador and Panama, and three (# 7, 25, 42) occur in Ecuador and/or Panama as well as North America (fig. 1).  

**Figure:**
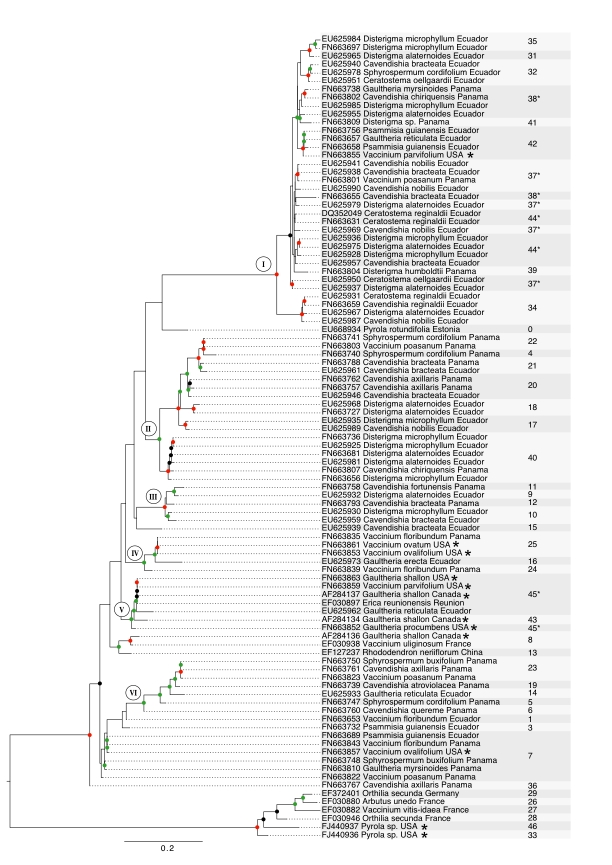

Figure 1: Phylogeny of Sebacinales associated with Ericaceae based on ITS data and inferred with Maximum Likelihood analysis. Colored dots on nodes indicate bootstrap support obtained from 1000 bootstrap replicates. Nodes with no dots are unsupported (below 50%), black dots indicate weakly supported clades (50-69%), green dots indicate moderately supported clades (70-89%) and red dots indicate well-supported clades (90-100%).  Each sequence label contains accession number, name of host species and country/locality where it has been sampled. Asterisks mark sequences from North America. Numbers indicate affiliation to Molecular Operational Taxonomic Units (MOTUs) obtained by cluster analysis and MOTUs with asterisks indicate MOTUs that are split into several phylogenetic clades. Gray background is used to enhance visibility of MOTU affiliation and has no other meaning beyond that. The tree was midpoint rooted.

**Figure:**
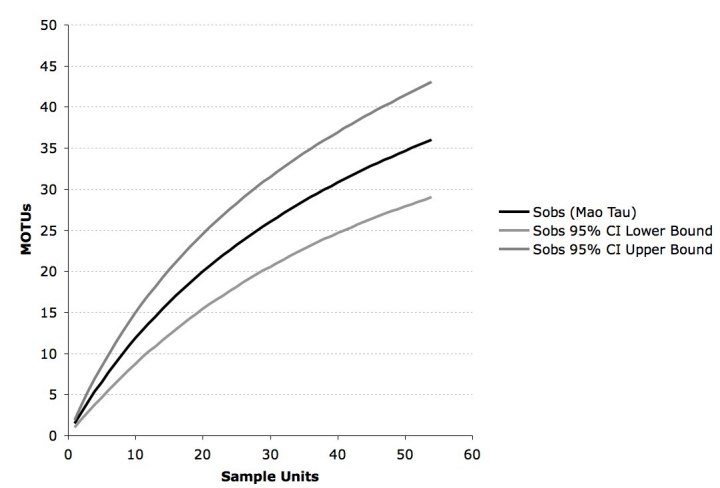

Figure 2: Rarefaction accumulation curve of all Molecular Operational Taxonomic Units (MOTUs) in our data set (Ecuador, Panama, USA) with 95% confidence intervals. Sobs = number of MOTUs observed by resampling without replacement.

## Discussion

Using the broadest sampling of Sebacinales from American Vaccinioideae to date, this study reveals that Sebacinales associated with Neotropical and North American Vaccinioideae are closely related. This is shown by the presence of several moderately to well-supported clades containing Sebacinales associated with both Neotropical and North American Vaccinioideae, even though most of the backbone is unsupported (fig. 1).

The species delimitation based on a threshold of 3% ITS1-ITS2 variability further shows that Sebacinales associated with Neotropical Vaccinioideae also occur in South, Meso- and North America (fig. 1). They are not restricted to Ecuador as previous studies with a more limited data set based on the D1/D2 region of the LSU indicated [7] [14]. The ITS region is more variable than the D1/D2 region of the LSU and allows phylogenetic resolution at a lower taxonomic level [19]. The 3% threshold of ITS variability was shown to be useful for species delimitation in basidiomycetes [19] [20] [21] and thus we have used the same threshold here to delimitate MOTUs and estimate the distribution range of Sebacinales associated with Neotropical and North American Vaccinioideae. If we had chosen a higher or lower threshold, we would have likely obtained a higher or lower number of widespread MOTUs, respectively.

Clades were inferred from phylogenetic analysis using the Maximum Likelihood principle and MOTUs were obtained by non-hierarchical linkage clustering based on a distance threshold. Because of these differences, MOTUs and clades are not necessarily in concordance. Here, most MOTUs do correspond to clades, but four MOTUs are placed in more than one clade, respectively (MOTUs # 37, 38, 44, 45 - fig. 1). Of these, MOTUs # 37, 38 and 44 are located in subclade I (fig. 1) which is a clade with low resolution. Thus, MOTUs and clades are not in real conflict with each other. In case of MOTU # 45, the split was caused by the sequence AF284143 (MOTU # 43) and we suppose that a less stringent clustering algorithm (e.g. single linkage clustering) would not have caused this conflict, since distances below the threshold would be required not for all, but only a proportion of sequences from the same cluster. In the future, it might be useful to delimit MOTUs with different linkage fractions in order to evaluate if there is an optimal linkage approach leading to a total congruence between MOTUs and clades.

The data set consists predominantly of Sebacinales from South and Mesoamerica and contains only few Sebacinales from North American Vaccinioideae. Considering the different species diversity of Vaccinioideae in the Neotropics (~800 species) and in North America (~60 species), the data set is relatively balanced with regard to host plant diversity. It is likely that the data accurately mirror the overall relationships of Sebacinales associated with Vaccinioideae from North America and the Neotropics. 

Regarding the distribution range, we expect that more extensive sampling of Sebacinales from North American Vaccinioideae would result in a higher number of widespread MOTUs rather than endemic ones, since all MOTUs from North America in this study also occur in other areas (fig. 1).

The occurrence of closely related Sebacinales associated with Vaccinioideae from North America, Meso- and South America supports the hypothesis of a coupled migration of ancestral Vaccinioideae and Sebacinales. If ancestral species dispersed to the Neotropics by long distance dispersal and associated with local Sebacinales, the extant Sebacinales from Neotropical and North American Vaccinioideae should be distantly related. A previous study investigating Sebacinales associated with coexisting Ericaceae and Orchidaceae from Southern Ecuador showed that each host family has different mycobionts [17] [18]. This corroborates our results inasmuch as it shows that the Sebacinales community in Southern Ecuador is complex and it indicates that Ericaceae have not adopted mycobionts at least from ancestral Orchidaceae.

The current theory about the origin of Neotropical Vaccinieae and Gaultherieae is that the ancestors were widely distributed and that diversification of Vaccinieae occurred during the late Miocene [1] [2]. If this is the case, the ancestors must have reached South America over the Central American Archipelago, because at that time a land bridge between the two subcontinents was not yet established. This indicates that stepping stone dispersal over islands played a role in the distribution of Vaccinioideae and their Sebacinales to South America. The question remains as to the mechanism by which plants and mycobionts managed to spread together. It is highly unlikely that dispersal occurred due to co-transport of seeds and spores, because it takes two compatible basidiospores to form a dicaryotic mycelium, colonize roots and successfully establish a mycorrhiza [4]. Furthermore, the mycorrhizal symbiosis maintains its stability by horizontal transmission of a variety of mycorrhizal fungi to the next plant generation [16]. Even though asexual spores might be a source of inoculation, Sebacinales form chlamydospores in the soil and not on aerial parts [34].

A possibility would be the dispersal of an entire plant with mycorrhizae and soil attached. That way a subset of mycorrhizal fungi could be transmitted at the same time, since different mycorrhizal fungi normally occur on the same root system [3] [4] [35]. Such a scenario would necessitate violent storms such as hurricanes that are known to have the potential to transport plants and even animals to distant islands [36]. Therefore, it is plausible that plants and mycobionts passively reached distant regions together. In addition, many Neotropical Vaccinioideae recover easily after disruption and are able to spread by adventitious roots [1] and establish in new habitats.

If Sebacinales associated with Neotropical Vaccinioideae are descendents of North American Sebacinales, we would expect to find North American Sebacinales in phylogenetically basal positions and Sebacinales from Neotropical Vaccinioideae in derived positions. Due to lacking backbone support, phylogenetic relationships of many major Sebacinales clades remain unresolved. However, on a lower taxonomic level the phylogeny gives insights into potentially derived and basal Sebacinales (fig. 1). There are three clades that contain Sebacinales of North American and Neotropical Vaccinioideae, but only one with Sebacinales from North America in a basal position (clade V). For clade IV it cannot be determined whether North American or Neotropical Sebacinales are basal or derived, since both have long branch lengths. Clade I contains one Sebacinales accession from North America (mycobiont of *Vaccinium parvifolium*) in a derived position (fig. 1). With exception of this taxon, all Sebacinales of clade I are from the Neotropics. Furthermore, this clade is derived and the branch lengths of Sebacinales within clade I are relatively short. If we assume a molecular clock in Sebacinales, the short branch lengths indicate diversification in a relatively short time period. Judging from this, it seems plausible that clade I originated in the Neotropics and the presence of one Sebacinales accession in North America is the result of a dispersal event out of the Neotropics into North America and not vice versa. This does not contradict the hypothesis of a coupled migration of ancestral Vaccinioideae and Sebacinales, but shows that putative re-migration events from the Neotropics to North America may have occurred. This should be taken into account especially since formation of the Central American landbridge about 3.5 million years ago facilitated dispersal in both directions, which led to the Great American Interchange [36]. To infer the ancestral symbiotic state of clade I, it would be highly valuable to compare Sebacinales from Neotropical Vaccinioideae to those from other coexisting hosts like liverworts and plants that might harbor endophytic Sebacinales [37] [31]. Adoption of ectomycorrhizal (e.g. *Sebacina incrustans*, *S. epigaea*) or saprophytic Sebacinales (species of the genera *Efibulobasidium* and *Craterocolla*) seems unlikely, since these fungi are only distantly related to Sebacinales of Vaccinioideae [29].

The results show that the evolutionary history of Sebacinales associated with American Vaccinioideae is complex and likely tied to the biogeography of their hosts. Here, we only focused on Sebacinales as mycorrhizal fungi of American Vaccinioideae, but it should be kept in mind that ascomycetes, especially Helotiales, also are important mycobionts of Vaccinioideae. We think that insights into the phylogeny of ascomyceteous mycobionts would be highly valuable and will help to further disentangle the complex evolutionary and geographic history of Neotropical Vaccinioideae and their mycorrhizal fungi.

## Acknowledgements

We thank Catherine Bush for mycorrhizal samples of some *Gaultheria* species and Jim Luteyn, Paola Pedraza, Ann Powell and Catherine Bush for help with species identification of Vaccinieae and Gaultherieae. We also thank Michael Weiß for kindly providing Sebacinales specific primers. We also want to thank Ingrid Kottke for many valuable comments and Tanja Schuster for critically revising earlier versions of this manuscript.

The support and assistance of Meike Piepenbring, Orlando Cáceres and the UNACHI in Panama, as well as of Juan Pablo Suárez, the UTPL and members of the research group FOR 816 in Ecuador are highly appreciated.

## Funding Information

The project was funded by the DFG as part of the research unit FOR 816 and by Wake Forest University, NC, USA.

## Competing interests

The authors have declared that no competing interests exist.

## Appendix

Table 2: Accession numbers and information for sequences downloaded from Genbank.

**Table:** 

**Accession**	**Organism**	**Host **	**Citation**
AF284134	uncultured Sebacinales	*Gaultheria shallon*	[6]
AF284136	uncultured Sebacinales	*Gaultheria shallon*	[6]
AF284137	uncultured Sebacinales	*Gaultheria shallon*	[6]
DQ352049	uncultured Sebacinales	*Ceratostema reginaldii*	[17]
EF030880	uncultured Sebacinales	*Arbutus unedo*	[7]
EF030882	uncultured Sebacinales	*Vaccinium vitis-idaea*	[7]
EF030897	uncultured Sebacinales	*Erica reunionensis*	[7]
EF030938	uncultured Sebacinales	*Vaccinium uliginosum*	[7]
EF030946	uncultured Sebacinales	*Orthilia secunda*	[7]
EF127237	uncultured Sebacinales	*Rhododendron neriflorum*	[7]
EF372401	Sebacinaceae sp. kz24	*Orthilia secunda*	[7]
EU668934	uncultured *Sebacina*	*Pyrola rotundifolia*	[38]
FJ440936	uncultured *Sebacina*	*Pyrola aphylla/picta*	[39]
FJ440937	uncultured *Sebacina*	*Pyrola aphylla/picta*	[39]
